# Genome-Scale Screening of *Saccharomyces cerevisiae* Deletion Mutants to Gain Molecular Insight into Tolerance to Mercury Ions

**DOI:** 10.3390/jof10070492

**Published:** 2024-07-16

**Authors:** Jianing Xian, Leilei Ni, Chengkun Liu, Jiyang Li, Yuhang Cao, Jie Qin, Dongwu Liu, Xue Wang

**Affiliations:** School of Life Sciences and Medicine, Shandong University of Technology, Zibo 255049, China; 23410011015@stumail.sdut.edu.cn (J.X.); 23510021033@stumail.sdut.edu.cn (L.N.); 18410370606@stumail.sdut.edu.cn (C.L.); 21110701008@stumail.sdut.edu.cn (J.L.); 18110701015@stumail.sdut.edu.cn (Y.C.); qinjie@sdut.edu.cn (J.Q.); liudongwu@sdut.edu.cn (D.L.)

**Keywords:** mercury, single-gene deletion mutants, genome-scale screen, detoxification, *Saccharomyces cerevisiae*

## Abstract

Mercury (Hg) is a global pollutant and a bioaccumulative toxin that seriously affects the environment. Though increasing information has been obtained on the mechanisms involved in mercury toxicity, there is still a knowledge gap between the adverse effects and action mechanisms, especially at the molecular level. In the current study, we screened a diploid library of *Saccharomyces cerevisiae* single-gene deletion mutants to identify the nonessential genes associated with increased sensitivity to mercury ions. By genome-scale screening, we identified 64 yeast single-gene deletion mutants. These genes are involved in metabolism, transcription, antioxidant activity, cellular transport, transport facilitation, transport routes, and the cell cycle, as well as in protein synthesis, folding, modification, and protein destination. The concentration of mercury ions was different in the cells of yeast deletion mutants. Moreover, the disruption of antioxidant systems may play a key role in the mercurial toxic effects. The related functions of sensitive genes and signal pathways were further analyzed using bioinformatics-related technologies. Among 64 sensitive genes, 37 genes have human homologous analogs. Our results may provide a meaningful reference for understanding the action mode, cellular detoxification, and molecular regulation mechanisms of mercury toxicity.

## 1. Introduction

Mercury (Hg) is a global pollutant that is among the most poisonous metals that adversely affect human health and the ecological environment [1]. The danger of its high toxicity, persistence, and widespread contamination has been given widespread attention since Minamata disease was identified as a severe case of mercury poisoning [2,3]. According to the World Health Organization, Hg exposure is not only associated with toxic effects on the immune, digestive, and nervous systems but also has adverse effects on the kidneys, lungs, eyes, and skin of humans.

It is known that the forms of Hg include elemental Hg (Hg^0^), inorganic Hg (Hg^+^, Hg^2+^), and organic Hg (RHg, R_2_Hg) [4]. Cases of metallic mercury poisoning usually result from mishandling of metallic mercury or contact with products containing metallic mercury. Thanks to the Minamata Convention and the automation of industrial production, the risk of mercury poisoning has been greatly reduced for the general public [5]. However, inorganic Hg, especially mercuric Hg (Hg^2+^), is abundantly found in the environment. Inorganic Hg usually undergoes methylation in the anaerobic environment and aquatic systems. Then, it changes into organic Hg, which is one of the most toxic forms of Hg. Organic mercury is mainly composed of short-chain alkyl mercury, which readily forms a complex with sulfhydryl substances and lipids on the cell membrane. The complex can not penetrate cells, but it is deposited around the cell wall and plasma membrane. The core of the complex contains mercury sulfide, and it can accumulate in the central nervous system, liver, and kidneys. Additionally, it inhibits the activities of T-ATPase, Mg^2+^-ATPase, and Na^+^ and K^+^-ATPase on the erythrocyte membrane and microsome membrane of the brain, liver, and kidneys. The complex significantly decreases the content of sulfhydryl in the cell membrane and brain microsome membrane, which results in changes in membrane conformation and function. Then cell growth is inhibited, and cell apoptosis and cell killing are further induced [6,7]. Moreover, Hg could be accumulated in aquatic animals and finally enter the human body as a food source. At present, it is the most common means of Hg exposure. Globally, methyl Hg has been detected in fish and other kinds of seafood [8], and humans may absorb Hg by eating different kinds of seafood. Therefore, it is crucial to uncover the molecular toxic mechanism of mercury and the cellular response mechanism that is induced by mercury.

Yeast is a eukaryotic model with similar structural characteristics and life activities to animals and plants. Molecular mechanisms and cell interactions with heavy metals can be easily studied in yeast [9]. Studies on the stress of heavy metal ions in yeast cells not only help to understand how cells regulate metal homeostasis but also contribute to identifying human homologous systems. In addition, it helps to diagnose and treat human diseases related to the destruction of ion homeostasis in cells. In this study, to fully understand the mechanism of mercury poisoning in eukaryotic cells, we screened mercury-sensitive genes in the whole genome of *Saccharomyces cerevisiae* by using a yeast deletion mutant library. Moreover, bioinformatics was used to analyze the classification and subcellular localization of mercury in yeast cells.

## 2. Materials and Methods

### 2.1. Strains and Media

The *S. cerevisiae* strains used in this study include wild-type BY4743 (*MATa/*α *ura3∆0*/*ura3∆0*, *his3∆1*/*his3∆1*, *leu2∆0*/*leu2∆0*, *LYS2/lys2∆0*, and *MET15*/*met15∆0*), BY4741 (*MATa his3Δ1; leu2Δ0; met15Δ0; ura3Δ0*), and a library of diploid single-gene deletion strains of *S. cerevisiae* with BY4743 as background (purchased from Invitrogen, Waltham, MA, USA). In addition, a solid-rich medium (YPD) (1% yeast extract, 2% glucose, and 2% peptone) was used to maintain yeast strains [10,11]. Mercuric chloride (HgCl_2_) was purchased from Sinopharm, Haidian District, Beijing, China.

### 2.2. Genome-Scale Genetic Screen for Mercury-Sensitive Mutations

Firstly, 200, 400, 600, and 800 μM Hg^2+^ were used to detect the toxicity of Hg^2+^ on yeast BY4743 and BY4741. Then, 100, 200, 300, and 400 μM Hg^2+^ were used to detect the toxicity of Hg^2+^ in yeast cells based on the previous study. Through preliminary experiments, we determined that the most suitable concentration for screening mercury-sensitive genes was 200 μM Hg^2+^. The preliminary screening of 4741 strains in the library was performed using the drop plate method to detect mercury ion sensitivity. The mutants in the library were dropped onto the YPD medium with or without 200 μM HgCl_2_ using a 384-pin replicator. The plates were incubated for 2 days at 30 °C, and the plates were photographed and used to analyze the growth of each individual mutant. The growth states of strains in the YPD plates containing HgCl_2_ were analyzed. Compared to the average size of its surrounding mutants, a mutant with a relative colony size reduced by more than 30% was identified as a mercury-sensitive mutant [12].

To clearly observe the differences in plaque growth phenotypes and the sensitivity of different individual mutant strains, the serial dilution method was used to verify the preliminary screening results. The mutant strains were inoculated from the mutant strain library into a liquid YPD medium for overnight growth. Then, it was diluted and spotted onto YPD plates with or without HgCl_2_. Based on the growth of their phenotype, their specific sensitivity to mercury was retested by comparing it with the wild-type strain BY4743 [13,14], and the secondary screen was repeated three times.

### 2.3. Measurement of Cellular Mercury Ion Content

The intracellular mercury content of the mutant strains sensitive to mercury was measured using an atomic fluorescence spectrometer (AFS-PF32). The yeast in the logarithmic growth phase was transferred to a YPD medium containing 100 μM HgCl_2_ for culture with shaking at 220 rpm and 30 °C for 4 h. Then, the fungi were washed, nitrated, and measured using atomic fluorescence spectrometry. Three individual colonies for each mutant were measured, and the wild-type BY4743 was used as a control in this assay.

### 2.4. Bioinformatic Analysis of the Deletion Mutant Data

The functions of the corresponding genes of mercury-sensitive mutants were annotated using the Saccharomyces Genome Database (SGD, http://www.yeastgenome.org, accessed on 1 Mrach 2022) and categorized based on biological function by using the GO Database. MIPS, BioGRID (http://www.thebiogrid.org, accessed on 1 March 2022), and FunSpec (http://funspec.med.utoronto.ca, accessed on 1 March 2022) were also used for annotation. The *p*-values that were calculated using a hypergeometric distribution represent the probabilities that the intersection of a given list with any given functional category occurs by chance.

### 2.5. Data Analysis

The result was expressed as the mean ± SEM. The difference in treatment groups was analyzed using SPSS 16.0 software. The one-way analysis of variance (ANOVA) was performed to assess the least significant difference (LSD), and *p* < 0.05 is set as the significance level.

## 3. Result

### 3.1. Genes Involved in the Hg^2+^ Sensitivity of Yeast Cells

**Figure 1:**
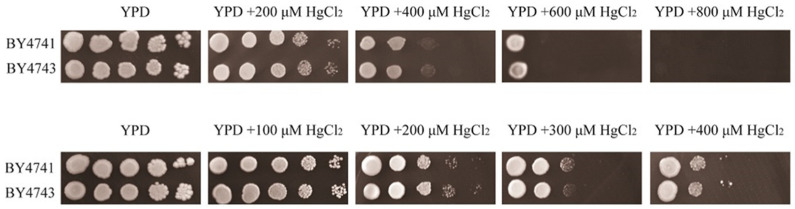
Determination of the screening concentration of Hg^2+^. In the first screening, the concentration of Hg^2+^ was 200, 400, 600, and 800 μM Hg^2+^. In the second screening, the concentration of Hg^2+^ was 100, 200, 300, and 400 μM Hg^2+^. The suitable concentration for screening mercury-sensitive genes was 200 μM Hg^2+^.

In this study, we first tested the sensitivity of the wild-type BY4743 strain to HgCl_2_. The result of gradients of HgCl_2_ on yeast cells showed that yeast cells had specific sensitivity at 200 μM HgCl_2_ (Figure 1). Thus, we used 200 μM HgCl_2_ to screen the yeast mutants.

**Figure 2:**
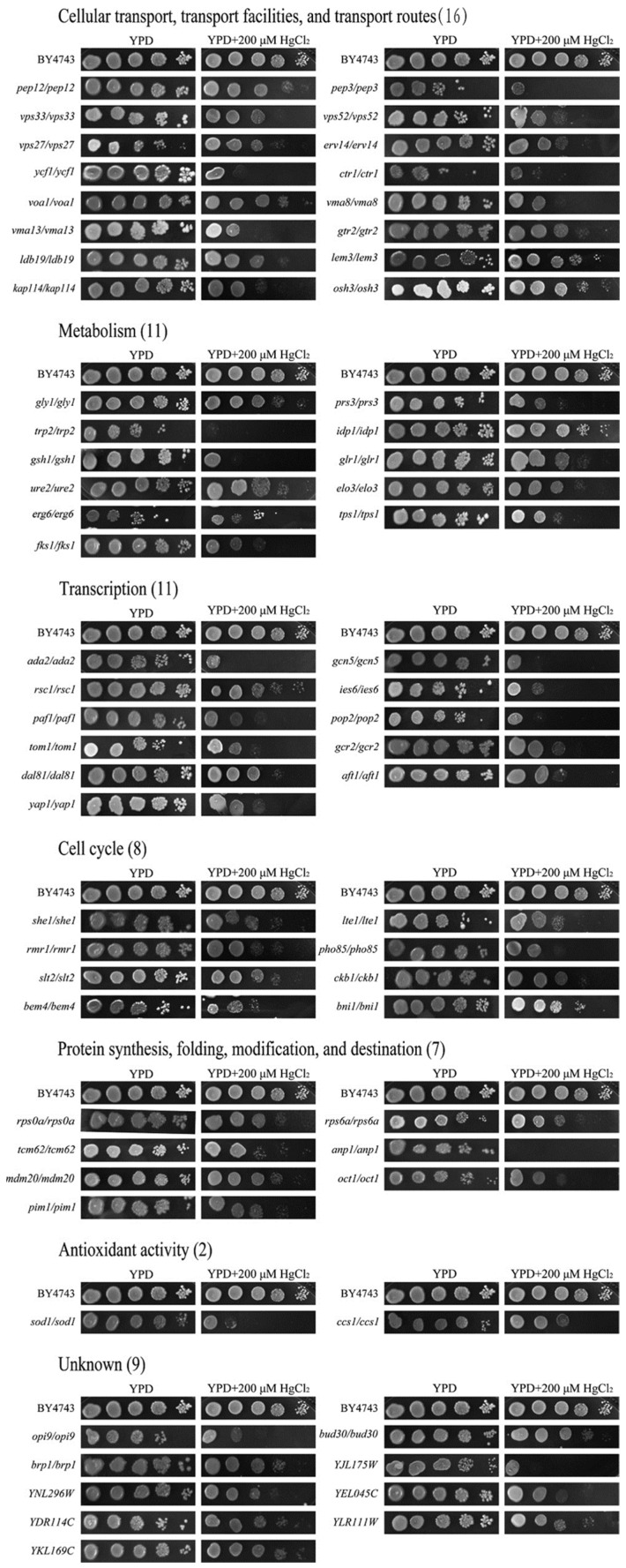
Phenotypes of Hg-sensitive gene deletion mutants. To identify the mercury-sensitive genes in *S. cerevisiae*, by observing and comparing the growth of the mutant strain exposed to mercury ions and the wild-type BY4743 strain, the difference in mutant growth states was compared. Finally, 64 gene deletion mutants were identified from the genome-scale screen.

Through a 384-pin replicator, a total of 374 strains sensitive to Hg^2+^ were preliminarily identified through screening of 4741 strains in the library. Then, a serial dilution method was used, and the sensitivity of strains to HgCl_2_ was scored by visual inspection of the imaged colonies. By observing and comparing the growth of mutant strains exposed to mercury ions and the wild-type BY4743 strain, we can clearly see the difference in mutant growth states (Figure 2). Finally, 64 nonessential gene deletion strains were further confirmed. Figure 2 shows that 64 genes were identified after the dilution of 374 strains.

After three repeated phenotypic experiments to verify the sensitive mutants, the genotypes of these deletion mutant strains were further confirmed by PCR with forward primers for their corresponding genes paired with primers KanMX4-F (Supplementary Table S1 and Supplementary Figure S1).

### 3.2. The Functional Classification and Subcellular Localization of Hg-Sensitive Genes

The functional classification shows that 64 mercury-sensitive genes can be divided into the following seven functional groups: cellular transport, transport facilities, and transport routes (16); metabolism (11); transcription (11); cell cycle (8); protein synthesis, folding, modification, and destination (7); antioxidant activity (2); and unclassified (9) (Table 1, Figure 2). Proteins encoded by these genes are mainly localized in the nucleus (12), vacuole (6), cytosol (20), endoplasmic reticulum (ER) (6), Golgi (2), mitochondrion (5), endosome (1), plasmalemma (2), and unknown genes (10) (Table 2). In addition, the functions of 64 mercury-sensitive genes were analyzed based on the Saccharomyces Genome Database (https://yeastgenome.org/, accessed on 1 March 2022), which are listed in Supplementary Table S2.

### 3.3. Measurement of Intracellular Mercury Content in Mercury Ion-Sensitive Mutants

**Figure 3:**
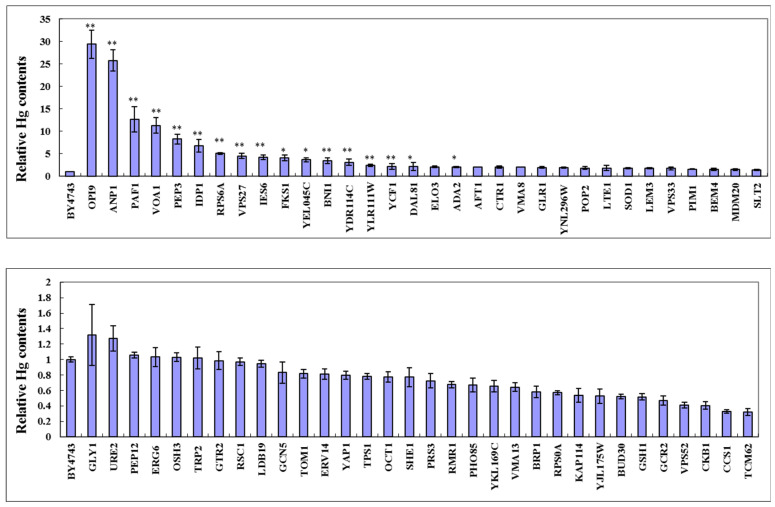
Cellular Hg contents of 64 Hg-sensitive gene deletion mutants in response to Hg stress. The intracellular mercury content of the mutant strains sensitive to mercury was measured using an atomic fluorescence spectrometer (AFS-PF32). The result was expressed as the mean ± SEM. The difference in treatment groups was analyzed using SPSS 16.0 software. The one-way analysis of variance (ANOVA) was performed to assess the least significant difference (LSD), and *p* < 0.05 is set as the significance level. ** *p* < 0.01, * *p* < 0.05.

The intracellular Hg^2+^ concentration of 64 mercury-sensitive strains was further detected. It is found that the mercury ion content in the two highly sensitive mutant strains of OPI9 and ANP1 is much higher than that of the wild-type, being about 29 and 25 times the control, respectively (Figure 3). The concentration of Hg^2+^ accumulated in the six deletion strains (*PAF1*, *VOA1*, *PEP3*, *IDP1*, *and RPS6A*) was 5~12 times higher than that of wild-type yeast (Figure 3). There are 23 sensitive strains (*VPS27*, *IES6*, *FKS1*, *YEL045C*, *BNI1*, *YDR114C*, *VMA8*, *AFT1*, *CTR1*, *YLR111W*, *YCF1*, *DAL81*, *ELO3*, *ADA2*, *GLR1*, *YNL296W*, *POP2*, *LTE1*, *SOD1*, *LEM3*, *VPS33*, *PIM1*, *BEM4*) with Hg^2+^ concentrations 2~4 times higher than those of the BY4743 strain (Figure 3). In addition, the mercury concentration in the strains *GCR2*, *VPS52*, *CKB1*, *CCS1*, and *TCM62* was 2~4 times lower than that of the BY4743 strain (Figure 3). However, there are 29 mutant strains with similar values to the wild-type, including *MDM20*, *SLT2*, *GLY1*, *URE2*, *PEP12*, *ERG6*, *OSH3*, *TRP2*, *GTR2*, *RSC1*, *LDB19*, *GCN5*, *TOM1*, *ERV14*, *YAP1*, *TPS1*, *OCT1*, *SHE1*, *PRS3*, *RMR1*, *PHO85*, *YKL169C*, *VMA13*, *BRP1*, *RPS0A*, *KAP114*, *YJL175W*, *BUD30*, and *GSH1* strains (Figure 3).

### 3.4. The Overlapping Rate of Genome-Wide Genetic Screening for Sensitivity to Different

#### Heavy Metals

**Figure 4:**
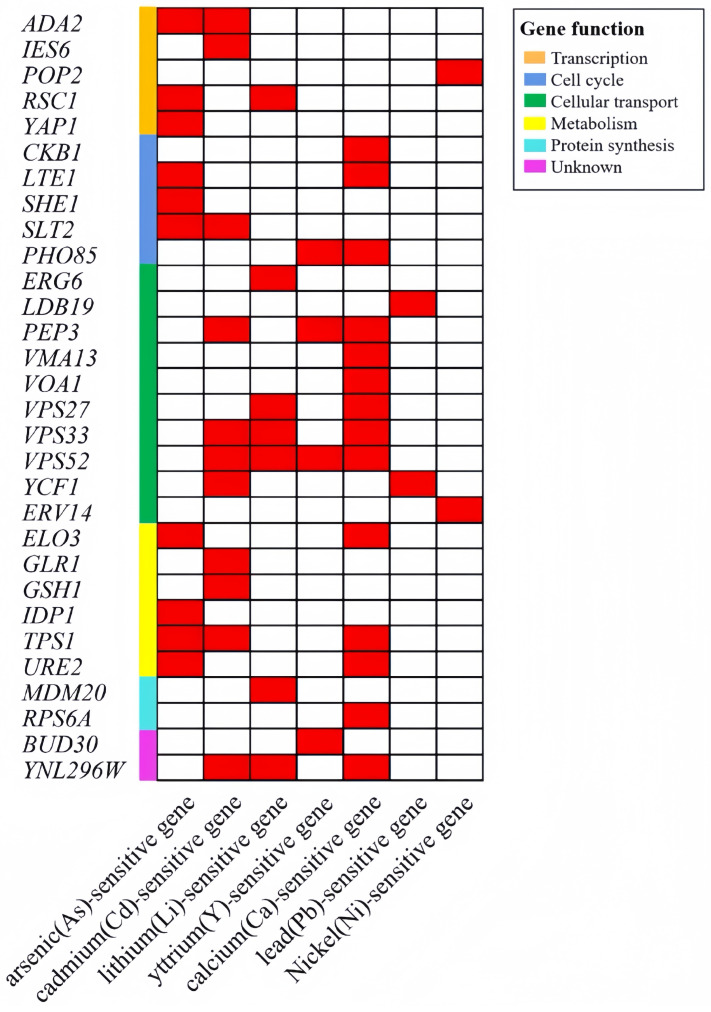
Overlap genes among heavy metal sensitive genes. Compared with the yeast cells sensitive to arsenic, cadmium, lithium, yttrium, calcium, lead, and nickel, the number of sensitive overlap genes was 10, 11, 7, 4, 14, 2, and 2, respectively.

Compared with the yeast cells sensitive to cadmium [13], lithium [15], yttrium [16], and calcium [14], the number of sensitive overlap genes was 11, 7, 4, and 14, respectively. For the 64 mercury-sensitive genes observed in this study, the overlap ratio with the four metal-sensitive genes mentioned above is 17.2% (11/64), 10.9% (7/64), 6.3% (4/64), and 21.9% (14/64), with an overlap rate between 6.3% and 21.9% (Figure 4). The relatively poor overlap may be due to the different mechanisms by which different metal ions exert toxic effects in cells, or it may be due to the different assay methods and different conditions used in the assay process. The shared genes between mercury-sensitive and cadmium, lithium, yttrium, and calcium-sensitive mutants are enriched in three categories, including cellular transport, transport facilitators, transport routes, metabolism, and cell cycle. Therefore, metabolism, cellular transport processes, and the cell cycle are all involved in the sensitivity of yeast cells to the stress of these metallic ions. However, an unknown gene, YNL296W, was found in the screening reports caused by Hg, Ca, Li, and Cd (Figure 4).

Additionally, arsenic is similar to mercury, tending to combine with sulfhydryl or disulfide to affect cell respiration and enzyme action and even cause chromosome breakage. Compared with the whole gene screening report for arsenic, there are 10 overlap genes, with an overlap rate of 15.6% (10/64) [17] (Figure 4). It is mainly concentrated in three categories, including metabolism, cell cycle, and transcription. In the screening of sensitive genes related to cadmium, lithium, yttrium, and calcium, some genes identified had roles in cellular transport, transport facilitation, and transport routes. However, these genes were not found in the arsenic screening results (Figure 4).

### 3.5. Bioinformatics Enrichment Analysis of Mercury-Sensitive Genes

Metascape was used to analyze the enrichment of the selected genes [18]. In terms of GO biological processes, 20 genes (*FKS1*, *YCF1*, *TOM1*, *VPS52*, *AFT1*, *SOD1*, *OCT1*, *DAL81*, *LEM3*, *BNI1*, *URE2*, *VPS27*, *GLR1*, *PHO85*, *VMA13*, *CTR1*, *PRS3*, *SLT2*, *VMA8,* and *GLY1*) participated in biological quality control, and 14 genes (*ADA2*, *VPS52*, *PAF1*, *AFT1*, *GTR2*, *GCN5*, *SOD1*, *LDB19*, *DAL81*, *YAP1*, *GCR2*, *POP2*, *PHO85*, and *OSH3*) participated in the positive regulation of cellular metabolic processes. Nine genes, including *VPS33*, *VPS52*, *PAF1*, *GTR2*, *PEP12*, *VPS27*, *PHO85*, *SLT2*, and *OSH3*, were involved in autophagy. There are three biological processes involving seven genes: response to inorganic substances (*YCF1*, *TPS1*, *GSH1*, *SOD1*, *YAP1*, *CCS1*, and *URE2*), regulation of transport (*PEP3*, *FKS1*, *VPS33*, *AFT1*, *LDB19*, *PHO85*, and *SLT2*), and positive regulation of cellular component organization (*FKS1*, *LTE1*, *ADA2*, *PAF1*, *LDB19*, *BNI1*, and *SLT2*). Five different genes are involved in post-Golgi vesicle-mediated transport (*PEP3*, *ELO3*, *VPS33*, *VPS52*, and *PEP12*) and the establishment or maintenance of cell polarity (*ERV14*, *BNI1*, *BEM4*, *PHO85*, and *OSH3*). In addition, a small number of mercury-sensitive genes also participated in the regulation of cell size (*FKS1*, *TOM1*, *PRS3*, and *SLT2*), response to metal ions (*YCF1*, *GSH1*, *YAP1*, and *URE2*), chromatin remodeling (*ADA2*, *RSC1*, *GCN5*, and *IES6*), regulation of DNA-binding transcription factor activity (*SOD1*, *URE2*, and *PHO85*), negative regulation of autophagy (*PAF1*, *GTR2*, and *PHO85*), positive regulation of transcription elongation from RNA polymerase II promoter (*PAF1*, *GCN5*, and *POP2*), late endosome to vacuole transport (*PEP3*, *ELO3*, and *VPS27*), and cellular carbohydrate biosynthetic process (*FKS1*, *TPS1*, and *PHO85*) (Table 3).

In the analysis of GO cellular components, some genes were related to the composition of three cell components: fungal-type vacuole membrane (*PEP3*, *VPS33*, *YCF1*, *VOA1*, *GTR2*, *PEP12*, *VMA13*, and *VMA8*), transferase complex (*FKS1*, *ADA2*, *TPS1*, *PAF1*, and *GCN5*, *MDM20*, *PHO85*, *PRS3*, and *ANP1*), and ATPase complex (*RSC1*, *LEM3*, and *IES6*), respectively (Table 4).

In the enrichment analysis of GO molecular functions, the enriched terms are antioxidant activity (*SOD1*, *CCS1*, *URE2*, and *GLR1*), transcription coregulator activity (*ADA2*, *GCN5*, *DAL81*, *URE2*, and *GCR2*), phospholipid binding (*PEP3*, *VPS33*, *ADA2*, *VPS27*, and *OSH3*), Ras GTPase binding (*LTE1*, *VPS52*, *KAP114*, and *BNI1*), ATPase activity and coupled to transmembrane movement of substances (*YCF1*, *VMA13*, and *VMA8*), transferase activity, and transferring hexosyl groups (*FKS1*, *TPS1*, and *ANP1*) (Table 5).

According to the bioinformatics analysis of the KEGG pathway [19], these genes play a significant role in five important pathways in yeast cells. To perform KEGG pathway enrichment analysis, we utilized the DAVID (Database for Annotation, Visualization, and Integrated Discovery) tool. They are glutathione metabolism (*IDP1*, *GSH1*, *URE2*, and *GLR1*), phagosome (*VPS27*, *VMA13*, and *VMA8*), autophagy (*PEP3*, *VPS33*, *PHO85*, and *SLT2*), MAPK signaling pathway (*FKS1*, *PAF1*, *BNI1*, and *SLT2*), and biosynthesis of amino acids (*IDP1*, *PRS3*, *GLY1*, and *TRP2*) (Table 6).

**Figure 5:**
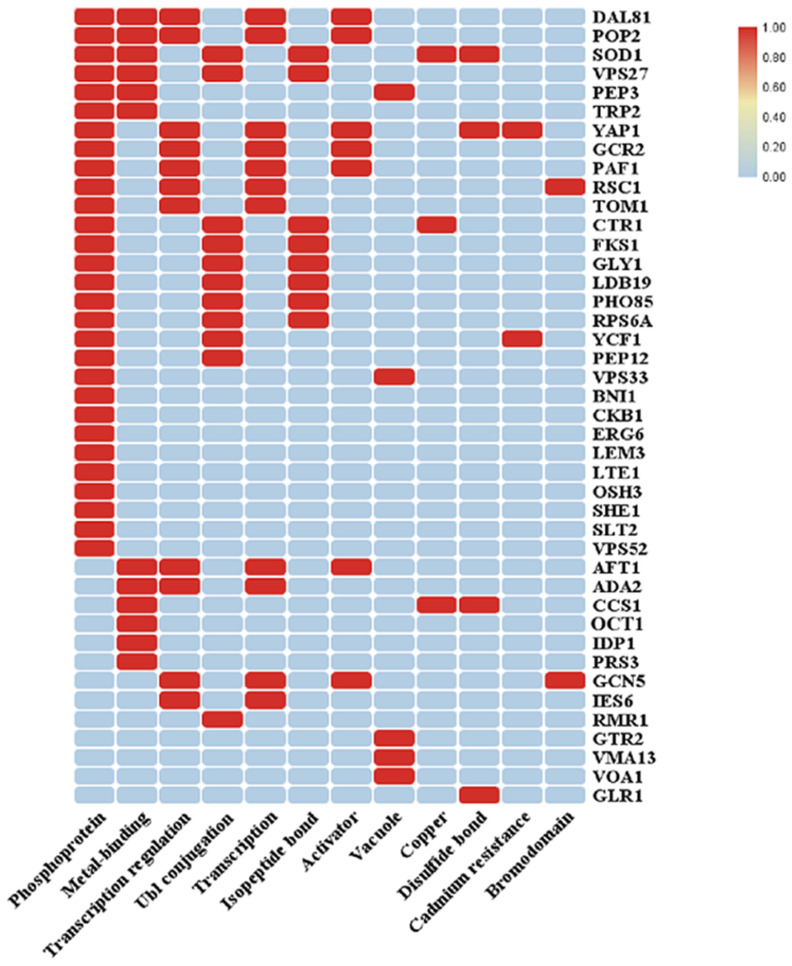
UP-keyword analysis of the Hg-sensitive gene (A).

**Figure 6:**
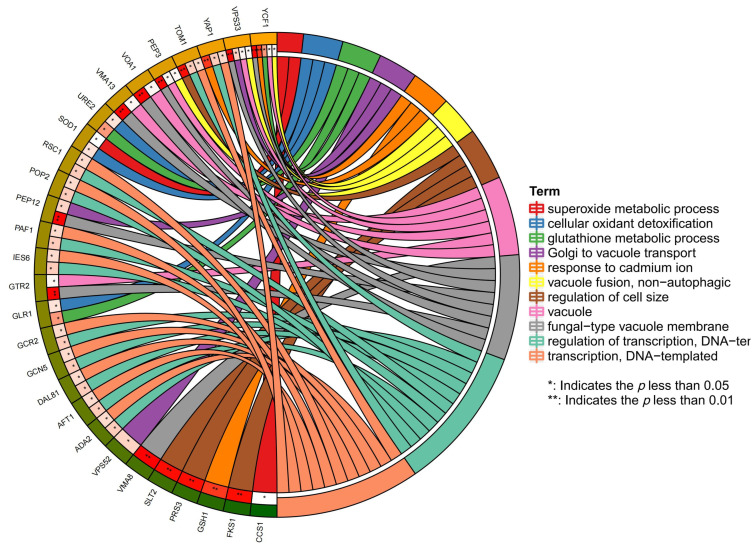
UP-keyword analysis of the Hg-sensitive gene (B).

Through the aforementioned method, we systematically revealed the enrichment characteristics of mercury-sensitive genes at the molecular function level, providing important references for further research into the functional mechanisms of these genes. UniProtKB Keywords (UP-keywords) constitute a controlled vocabulary with a hierarchical structure. Keywords summarize the content of a UniProtKB entry and facilitate the search for proteins of interest by analyzing DAVID [20]. The results of the category as UP-keywords showed that the top three *p*-value rankings were activator, transcription regulation, phosphoprotein, etc. (Table 7). To better show the correlation between different terms and genes, an analysis was performed on the data using Cytoscape [21] (Figure 5 and Figure 6).

### 3.6. The Result of Protein–Protein Interaction

In addition, protein–protein interaction analysis (PPI) refers to the process in which two or more protein molecules form protein complexes through non-covalent bonds. In cells, many protein elements combine with each other to form molecular machines. Through interaction between these combined proteins, many important processes related to growth and reproduction in cells are completed. Protein–protein interaction is the foundation of biochemical functions involved in normal cellular activities. Therefore, it is necessary to reveal the protein–protein interactions within yeast cells by studying mercury-sensitive genes and utilizing bioinformatics techniques.

**Figure 7:**
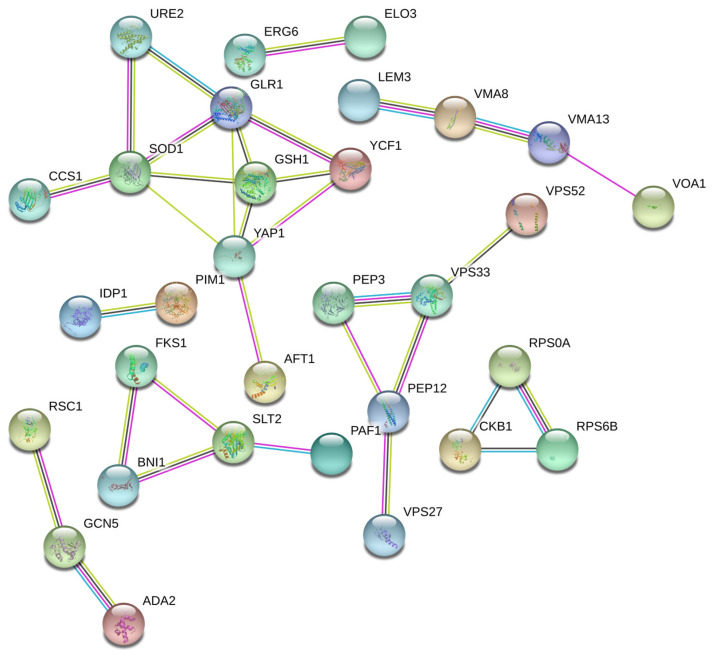
Protein–protein interaction network is formed by Hg-sensitive genes. In a network diagram, the nodes represent various different proteins, and the node labels are the names of these proteins. The pattern in the node represents the three-dimensional structure of the protein. If it is empty, it indicates that the structure is currently unknown. If there is an interaction between two proteins, they are connected by a line, and a thicker line shows a stronger interaction. The color of the connecting line reflects the type of interaction, including experimentally validated or predicted, as well as direct physical interactions, co-expression, gene fusion, and other relationships.

The STRING website was used to process the protein–protein interaction data on the screened genes [22]. The protein–protein interaction network was explored using Cytoscape [21]. In a network diagram, the nodes represent various different proteins, and the node labels are the names of these proteins (Figure 7). The pattern in the node represents the three-dimensional structure of the protein. If it is empty, it indicates that the structure is currently unknown. If there is an interaction between two proteins, they are connected by a line, and a thicker line shows a stronger interaction (Figure 7). The color of the connecting line reflects the type of interaction, including experimentally validated or predicted, as well as direct physical interactions, co-expression, gene fusion, and other relationships. The protein–protein interaction data on the screened genes are shown in Figure 7.

## 4. Discussion

In this study, we found that the deletion of 64 genes makes yeast cells sensitive to mercury stress. The proteins encoded by these genes are involved in numerous cellular processes, including metabolism, cell cycle, transcription, protein synthesis, folding, modification and destination, antioxidant activity, cellular transport, transport facilitation, and transport routes. The largest gene category (16 genes) is involved in cellular transport, transport facilitation, and transport routes. In contrast, the largest category that has been identified in lithium, lead, and cadmium-sensitive screens is also the cell transport process. It indicates that cell transport processes are of vital importance to the detoxification of various heavy metal ions such as mercury, lithium, lead, and cadmium in *S. cerevisiae* cells.

Vacuolar H^+^-ATPase (V-ATPase) is a multi-subunit complex composed of hydrophilic V1 and hydrophobic V0 complexes [23]. They have 8 and 6 subunits, respectively, and v-ATPase is responsible for ATP hydrolysis or proton translocation. The deletion of *VMA8* (encoding the D subunit of the V1 peripheral membrane domain), *VMA13* (encoding the H subunit of the V1 peripheral membrane domain), and *VOA1* (encoding the ER protein that functions in the assembly of the V0 sector) causes yeast cells to be sensitive to mercury stress [24,25]. Therefore, V-ATPase is essential for yeast cells to respond to mercury stress. The fusion of intracellular transport vesicles requires the soluble N-ethylmaleimide-sensitive factor attachment protein receptors (SNAREs) and Sec1/Munc18-family (SM) proteins. Membrane-bridging SNARE complexes are critical for fusion, but their spontaneous assembly is inefficient and may require SM proteins in vivo. In a previous study, it was reported that Vps33 and potentially other SM proteins could thus act as templates for generating partially zipped SNARE assembly intermediates. HOPS was essential to mediate SNARE complex assembly at physiological SNARE concentrations. Thus, Vps33 appears to catalyze SNARE complex assembly through specific SNARE motif recognition [26]. Pep12p was a target membrane receptor (t-SNARE) for vesicular intermediates traveling between the Golgi apparatus and the vacuole [27]. Vps52p is involved in the formation of the GARP (Golgi-associated retrograde protein) complex [28]. These three proteins are involved in the transport of Golgi to vacuoles. As part of the CORVET membrane tethering complex (class C core vacuole/endosome tethering), Pep3p promotes vesicular docking/fusion reactions in conjunction with SNARE proteins. Vps27p encodes representative subunits of the endosomal sorting complex required for transport (ESCRT) machinery in *S. cerevisiae*. Both genes participate in the transport process from the late endosome to the vacuole [29,30]. Ycf1 is a vacuolar glutathione S-conjugate transporter. At the same time, it also participates in the resistance to stress caused by metal ions such as lead, cadmium, and arsenite [31]. Furthermore, the deletion of genes *ERV14*, *CTR1, LEM3,* and *OSH3* causes cell transport disorder due to the involvement of membrane systems such as COPII-coated vesicle protein, high-affinity copper transporter of the plasma membrane, membrane protein of the plasma membrane, and ER, and interacts with ER anchor Scs2p at patches of the plasma membrane [32]. In addition, the deletion mutant of *ERG6* is sensitive to mercury, and we speculate that the hindrance of the synthesis of ergosterol is the reason why yeast is affected in material transportation. The endosomal sorting complex required for transport (ESCRT) negatively regulates Erg6p degradation under specific glucose restriction conditions. The transporting complex is the endosomal sorting complex required for transport (ESCRT). The endosomal sorting the complex required for transporting complex negatively regulates Erg6p degradation under specific glucose restriction conditions. *ERG6* also encodes Delta(24)-sterol C-methyltransferase and converts zymosterol to fecosterol in the ergosterol biosynthetic pathway by methylating position C-24 [30]. It is worth noting that ergosterol is an important component of microbial cell membranes. It also plays an important role in ensuring cell viability, membrane fluidity, membrane-bound enzyme activity, membrane integrity, and cellular material transport. It is well known that inorganic mercury can be converted into methyl mercury in organisms. And methylmercury is a kind of sulfhydryl-philic substance that can be combined with sulfhydryl-containing substances to form a mercury thiolate complex in the body. Thereby, it interferes with SH-related metabolism and leads to cell damage. The cell membrane is rich in -SH groups, which readily combine with methylmercury. This results in changes in membrane structure and function, reducing the fluidity in the membrane and enhancing permeability. Damage to the biofilm formation is central to the toxic mechanism of mercury (methylmercury) [33], which is consistent with our research results.

At present, it is believed that the main toxicological mechanism of mercury is the strong binding of mercury with macromolecules containing sulfhydryl and selenium (- SeH), such as glutathione. The binding destroys the biological functions of important molecules. Glutathione (GSH) is a highly effective antioxidant. It has a high mercaptan content and is abundant in yeast cells, which play an important role in antioxidant metabolism. In our screening, genes related to the synthesis and metabolism of glutathione (*GSH1*, *YAP1*, *GLR1*, *URE2*, *IDP1*, etc.) were identified as Hg-sensitive. Glutathione in yeast is synthesized by γ-GCS (γ-glutamylcysteine synthetase) and glutathione synthetase, which are encoded by *GSH1* and *GSH2*, respectively. γ-GCS is a key enzyme for glutathione synthesis. The expression of γ-GCS is highly regulated. In addition to feedback inhibition by the final product glutathione, it is also controlled by two regulators at the transcription level, namely Yap1p and Skn7p. Once stimulated by environmental changes, these two transcription factors bind to the promoter of *GSH1* and regulate the mRNA transcription of two subunits of γ-GCS, thus regulating the level of glutathione in cells [34,35].

Previously, studies have shown that the toxicity of Hg^2+^ is closely related to a decrease in the activity of free radical scavenging systems such as superoxide dismutase, glutathione peroxidase, etc. Glr1p is a cytoplasmic and mitochondrial glutathione oxidoreductase that can convert oxidized glutathione into reduced glutathione. Previous research has shown that oxidative stress causes the conversion of GSH to oxidized glutathione (GSSG), and the latter is reduced to GSH and NADP^+^ under the action of glutathione reductase and NADPH. Thus, it could reduce oxidative stress and restore the dynamic redox balance. The *GLR1* deletion mutant was sensitive to mercury, indicating that this process is also involved in the response to mercury stress. Moreover, Ure2p has glutathione peroxidase activity and can be mutated to obtain GST activity [36]. *SOD1* encodes cytoplasmic copper–zinc superoxide dismutase. Under oxidative stress, it enters the nucleus via Dun1p phosphorylation and promotes the transcription of stress-related genes. *CCS1* encodes a copper chaperone for the superoxide dismutase Sod1p, which is involved in oxidative stress protection. The Met-X-Cys-X2-Cys motif within the N-terminus is involved in the insertion of copper into Sod1p under conditions of copper deprivation [37,38]. It has been observed that mercury in the body binds to antioxidants such as glutathione (GSH) and inhibits the activity of anti-peroxidase systems such as GST and SOD, which reduces the ability to eliminate free radicals.

Mercury can also directly induce mitochondrial oxidative damage, leading to the accumulation of oxygen-free radicals (ROS). Since mitochondria are the main production site and target of reactive oxygen species, the accumulation of functionally defective mitochondria in cells will further lead to cell oxidative damage during oxidative stress. Therefore, it can be seen from the results that *TCM62* (encoding a protein involved in the assembly of the succinate dehydrogenase complex [39]), *MDM20* (involved in mitochondrial inheritance and actin assembly [40]), *OCT1* (encoding mitochondrial intermediate peptidase [41]), and *PIM1* (involved in the degradation of misfolded proteins in mitochondria [42]) are all sensitive to mercury ions.

Nucleic acid and protein are the core molecules of all life processes. Nucleic acid, as a template for protein construction, is an important molecule for storing biological information [43]. The inhibitory effect of mercury compounds on DNA and RNA synthesis has long been reported. A decrease in nucleic acid content may be attributed to the damage of free radicals to DNA and the inhibition of RNA synthesis by the direct action of ROS [44,45]. Meanwhile, in yeast cells, chromatin remodeling factors regulate chromatin structure by changing the assembly, disassembly, and rearrangement of nucleosomes on chromatin, thereby improving the local accessibility of transcription-related factors such as transcription factors in chromatin DNA. Under the action of the chromatin remodeling factor, when the chromatin structure tends to become looser, the accessibility of RNA polymerase II and transcription factor to chromatin DNA increases, thus initiating gene transcription. Conversely, when the chromatin structure tends to be dense, the accessibility of RNA polymerase II and transcription factors to chromatin DNA is weakened, thus inhibiting the transcription of related genes. Among the sensitive mutants, we found that Ada2p (transcription coactivator; component of the ADA and SAGA transcriptional adaptor/histone acetyltransferase complexes), Gcn5p (catalytic subunit of ADA and SAGA histone acetyltransferase complexes), Rsc1p (a component of the RSC chromatin remodeling complex), and Ies6p (component of the INO80 chromatin remodeling complex) were involved in the process of chromatin remodeling and sensitivity to mercury [46,47,48]. Gcn5p, Paf1p, and Pop2p are involved in the positive regulation of transcription elongation from the RNA polymerase II promoter. Ada2p and Gcr2p are involved in the transcriptional activation of RNA polymerase II [49]. Therefore, we infer that the transcriptional toxicity of mercury in yeast cells is related to the chromatin remodeling factor and RNA polymerase II.

Based on the growth phenotype of the mutant, we can see that there is a deletion mutant that is worthy of attention. The *ANP1* deletion mutant grew normally in the YPD medium. However, no signs of strain growth can be seen in the mercury-containing medium. The concentration of intracellular mercury was 29 times higher than that of the wild-type. This indicated that the mutant was extremely sensitive to mercury. When exposed to mercury ion stress, a large number of mercury ions enter cells, which poses a great threat to the growth of mutants. Anp1p is known to be a component of the α-1,6-mannosyltransferase complex and to be involved in the N-linked glycosylation of proteins. The mutant showed a reduced ability to form biofilm [50]. Therefore, we speculate that when *ANP1* is missing, mannose synthesis breaks off, the cell wall is incomplete, and the ability to form biofilm is decreased. Therefore, the mutant does not form an effective barrier against the influx of mercury ions, and the invading mercury ions display a powerful toxic effect.

In addition, two genes with unknown functions, *OPI9* and *YNL296W*, are also worth noting. *OPI9* is a dubious open reading frame. Based on the available experimental and comparative sequence data, it is unlikely to encode a functional protein. However, it was found that the intracellular ion concentration of the *OPI9-*deficient strain was 25 times that of the wild-type. Unlike the *ANP1* deletion strain, the *OPI9* deletion strain is not very sensitive. Therefore, the specific function of the *OPI9* gene deserves further exploration in the future. *YNL296W* is a dubious open reading frame that cannot encode a functional protein, and its deletion is unfavorable to spore formation. *YNL296W* was identified during the genome-wide screening of *S. cerevisiae* deletion mutants for sensitivity to Hg, Cd, Li, and Ca. Therefore, we infer that *YNL296W* may play a similar and positive role in the protection of *S. cerevisiae* from metal ions.

## 5. Conclusions

In summary, we employed a genome-wide high-throughput screen to elucidate the global response mechanism of yeast cells facing mercury ion stress. Bioinformatics methods were used to integrate data to further explore the signaling pathways and biological processes by which *S. cerevisiae* cells respond to mercury stress. By genome-scale screening, we identified 64 yeast single-gene deletion mutants. These genes are involved in metabolism, transcription, antioxidant activity, cellular transport, transport facilitation, transport routes, and the cell cycle, as well as in the processes of protein synthesis, folding, modification, and protein destination. Among the 64 sensitive genes, 37 have human homologous analogs. Our findings may provide clues for a deeper understanding of the toxicological mechanism of mercury in eukaryotic cells. It may help to elucidate the pathological processes related to mercury poisoning in human diseases.

## Figures and Tables

**Table 1:** Functional categories of 64 genes whose deletion mutants are sensitive to Hg^2+^.

Systemic Name	Standard Name	Systemic Name	Standard Name	Systemic Name	Standard Name	Systemic Name	Standard Name
Cellular transport, transport facilitators, and transport routes (16)							
YDR135C	*YCF1*	YGL241W	*KAP114*	YLR148W	*PEP3*	YOR036W	*PEP12*
YDR484W	*VPS52*	YGR106C	*VOA1*	YLR396C	*VPS33*	YOR322C	*LDB19*
YEL051W	*VMA8*	YGR163W	*GTR2*	YNL323W	*LEM3*	YPR036W	*VMA13*
YGL054C	*ERV14*	YHR073W	*OSH3*	YNR006W	*VPS27*	YPR124W	*CTR1*
Metabolism (11)							
YBR126C	*TPS1*	YER090W	*TRP2*	YLR342W	*FKS1*	YNL229C	*URE2*
YDL066W	*IDP1*	YHL011C	*PRS3*	YLR372W	*ELO3*	YPL091W	*GLR1*
YEL046C	*GLY1*	YJL101C	*GSH1*	YML008C	*ERG6*		
Transcription (11)							
YBR279W	*PAF1*	YEL044W	*IES6*	YGR252W	*GCN5*	YNL199C	*GCR2*
YDR448W	*ADA2*	YGL071W	*AFT1*	YIR023W	*DAL81*	YNR052C	*POP2*
YDR457W	*TOM1*	YGR056W	*RSC1*	YML007W	*YAP1*		
Cell cycle (8)							
YBL031W	*SHE1*	*YGL250W*	*RMR1*	YHR030C	*SLT2*	YPL161C	*BEM4*
YAL024C	*LTE1*	*PHO85*	*PHO85*	YGL019W	*CKB1*	YNL271C	*BNI1*
Protein synthesis, folding, modification, and destination (7)							
YBL022C	*PIM1*	YEL036C	*ANP1*	YKL134C	*OCT1*	YPL090C	*RPS6A*
YBR044C	*TCM62*	YGR214W	*RPS0A*	YOL076W	*MDM20*		
Antioxidant activity (2)							
YJR104C	*SOD1*	YMR038C	*CCS1*				
Unknown (9)							
YDL151C	*BUD30*	YGL007W	*BRP1*	YKL169C		YLR338W	*OPI9*
YDR114C		YJL175W		YLR111W		YNL296W	
YEL045C							

**Table 2:** The subcellular localization of 64 genes whose deletion mutants are sensitive to Hg^2+^.

Cellular Localization	Gene
cytosol (20)	*GCN5, POP2, AFT1, YAP1, LTE1, RMR1, SLT2, LDB19, LEM3, KAP114, RPS0A, SOD1, CCS1, GLY1, PRS3, TRP2, GSH1, GLR1, URE2, TPS1,*
nucleus (12)	*ADA2, RSC1, IES6, PAF1, TOM1, GCR2, DAL81, SHE1, PHO85, BEM4, CTR1, RPS6A,*
vacuole (6)	*PEP3, VPS33, YCF1, VMA8, VMA13, GTR2,*
endoplasmic reticulum (ER) (6)	*ERV14, VOA1, OSH3, ANP1, ELO3, ERG6,*
mitochondrion (5)	*TCM62, MDM20, OCT1, PIM1, IDP1,*
Golgi (2)	*PEP12, VPS52*
plasmalemma (2)	*BNI1, FKS1,*
endosome (1)	*VPS27*
unknown (10)	*CKB1, OPI9, BUD30, BRP1, YJL175W, YNL296W, YEL045C, YDR114C, YLR111W, YKL169C*

**Table 3:** GO biological processes enrichment analysis of Hg-sensitive genes.

Function	Number	Log10 (*p*)	Genes
Biological quality control	20	−6.86	*FKS1*, *YCF1*, *TOM1*, *VPS52*, *AFT1*, *SOD1*, *OCT1*, *DAL81*, *LEM3*, *BNI1*, *URE2*, *VPS27*, *GLR1*, *PHO85*, *VMA13*, *CTR1*, *PRS3*, *SLT2*, *VMA8*, *GLY1*
Response to inorganic substances	7	−5.59	*YCF1*, *TPS1*, *GSH1*, *SOD1*, *YAP1*, *CCS1*, *URE2*
Transportation regulation	7	−4.27	*PEP3*, *FKS1*, *VPS33*, *AFT1*, *LDB19*, *PHO85*, *SLT2*
Regulation of DNA-binding transcription factor activity	3	−3.9	*SOD1*, *URE2*, *PHO85*
Autophagy	9	−3.8	*VPS33*, *VPS52*, *PAF1*, *GTR2*, *PEP12*, *VPS27*, *PHO85*, *SLT2*, *OSH3*
Cell size regulation	4	−3.79	*FKS1*, *TOM1*, *PRS3*, *SLT2*
Response to metal ions	4	−3.79	*YCF1*, *GSH1*, *YAP1*, *URE2*

**Table 4:** GO cellular component enrichment analysis of Hg-sensitive genes.

Function	Number	Log10 (*p*)	Genes
Fungal-type vacuolar membrane	8	−2.93	*PEP3*, *VPS33*, *YCF1*, *VOA1*, *GTR2*, *PEP12*, *VMA13*, *VMA8*
Transferase complex	9	−1.9	*FKS1*, *ADA2*, *TPS1*, *PAF1*, *GCN5*, *MDM20*, *PHO85*, *PRS3*, *ANP1*
ATPase complex	3	−1.57	*RSC1*, *LEM3*, *IES6*

**Table 5:** GO molecular function enrichment analysis of Hg-sensitive genes.

Function	Number	Log10 (*p*)	Genes
Antioxidant activity	4	−3.68	*SOD1*, *CCS1*, *URE2*, *GLR1*
Transcription coregulator activity	5	−2.91	*ADA2*, *GCN5*, *DAL81*, *URE2*, *GCR2*
Phospholipid binding	5	−2.49	*PEP3*, *VPS33*, *ADA2*, *VPS27*, *OSH3*
Ras GTPase binding	4	−2.3	*LTE1*, *VPS52*, *KAP114*, *BNI1*
ATPase activity, coupled with the transmembrane movement of substances	3	−1.6	*YCF1*, *VMA13*, *VMA8*
Transferase activity, transferring hexosyl groups	3	−1.38	*FKS1*, *TPS1*, *ANP1*

**Table 6:** KEGG pathway enrichment analysis of Hg-sensitive genes.

Function	Number	Log10 (*p*)	Genes
Glutathione metabolism	4	−4.18	*IDP1*, *GSH1*, *URE2*, *GLR1*
Phagosome	3	−2.41	*VPS27*, *VMA13*, *VMA8*
Autophagy	4	−2.1	*PEP3*, *VPS33*, *PHO85*, *SLT2*
Signaling pathway	4	−1.65	*FKS1*, *PAF1*, *BNI1*, *SLT2*
Biosynthesis of amino acids	4	−1.55	*IDP1*, *PRS3*, *GLY1*, *TRP2*

**Table 7:** UP-keyword analysis of Hg-sensitive genes.

Function	Number	Log10 (*p*)	Genes
Activator	7	0.007	*GCR2*, *GCN5*, *DAL81*, *PAF1*, *AFT1*, *POP2*, *YAP1*
Transcription regulation	11	0.011	*TOM1*, *ADA2*, *GCR2*, *GCN5*, *DAL81*, *RSC1*, *PAF1*, *AFT1*, *IES6*, *POP2*, *YAP1*
Phosphoprotein	29	0.012	*TOM1*, *LTE1*, *VPS52*, *VPS27*, *DAL81*, *LEM3*, *PEP12*, *PEP3*, *ERG6*, *VPS33*, *YAP1*, *CTR1*, *SHE1*, *PAF1*, *RPS6A*, *LDB19*, *GCR2*, *BNI1*, *TRP2*, *PHO85*, *FKS1*, *RSC1*, *YCF1*, *GLY1*, *POP2*, *SLT2*, *CKB1*, *OSH3*, *SOD1*
Ubl conjugation	10	0.019	*RMR1*, *PHO85*, *FKS1*, *VPS27*, *GLY1*, *RPS6A*, *PEP12*, *LDB19*, *CTR1*, *SOD1*
Transcription	11	0.021	*TOM1*, *ADA2*, *GCR2*, *GCN5*, *DAL81*, *RSC1*, *PAF1*, *AFT1*, *IES6*, *POP2*, *YAP1*
Vacuole	6	0.024	*VMA13*, *YCF1*, *VPS33*, *PEP3*, *GTR2*, *VOA1*
Copper	3	0.029	*CCS1*, *CTR1*, *SOD1*
Disulfide bond	4	0.048	*CCS1*, *GLR1*, *YAP1*, *SOD1*
Cadmium resistance	2	0.055	*YCF1*, *YAP1*
Metal-binding	12	0.075	*ADA2*, *OCT1*, *TRP2*, *CCS1*, *VPS27*, *DAL81*, *AFT1*, *IDP1*, *POP2*, *PRS3*, *PEP3*, *SOD1*
Bromodomain	2	0.089	*GCN5*, *RSC1*
Isopeptide bond	8	0.095	*PHO85*, *FKS1*, *VPS27*, *GLY1*, *RPS6A*, *LDB19*, *CTR1*, *SOD1*

## Data Availability

The data obtained during and/or analyzed during the current study are available from the corresponding author upon reasonable request.

## Supplementary Materials

The following supporting information can be downloaded at: https://www.mdpi.com/article/10.3390/jof10070492/s1, Figure S1: Genotype verification of sensitive deletion strains; Table S1: PCR Primers used in this study; Table S2: The protein function and subcellular localization of 64 genes sensitive to Hg^2+^.
