# Supplementary material for: Genome-Scale Screening of Saccharomyces cerevisiae Deletion Mutants to Gain Molecular Insight into Tolerance to Mercury Ions
Source: J Fungi (Basel). 2024 Jul 16;10(7):492. doi: 10.3390/jof10070492 (PMC11277898; doi:10.3390/jof10070492)
Supplement: Supplementary file 1 [file jof-10-00492-s001.zip › jof-3044193-supplementary.pdf]

Supplementary tables and figures

Table S1. PCR Primers used in this study.

Primer	Sequence (5′–3′)	Primer	Sequence (5′–3′)
KanMX4-R	GCCGTTTCTGTAATGAAGGAG	*VPS33*-F	GCTGATATTGCCCATCTCC
ERG6-F	CTCCAATACTTGCTGTTGCC	*PEP3*-F	GCGCTTTCTGATTCTCTTCG
BEM4-F	GGCTGAAGACAACGTAAGC	*YLR111W*-F	CTGCCGTTATGTTGGAAGTAAG
YCF1-F	GTAACTCCTGGTGTGATGC	*GLR1*-F	CATGCGCTTCTCACTCTC
GLY1-F	CAAGTGTTGAGCTTGCCTG	*IDP1*-F	CCTTCAAGCAATTGTGAGAC
GTR2-F	TAATCATGCTACTGCTTCACC	*BNI1*-F	CGTCTCATACAGTTGGGC
RPS0A-F	GGATACCAGAATCCCGTCG	*TPS1*-F	CAAGCACGCAGCTAAGTAAG
RPS6A-F	CTCCTGGTAAGCGTTGGAG	*IES6*-F	GACGGTGAGGAATGGAAG
KAP114-F	GGATTCAGAAACTACCTCACA	*VMA13*-F	CTACCTACCCGTGCATAATTAC
PIM1-F	GATGTTTGCCACCGGAAG	*MDM20*-F	GCAACGATACATAACGAGC
RMR1-F	GCAAAGTCTAAATACCCGCC	*BUD30*-F	GACAGTTTCAACGTCTTCG
SHE1-F	TAAACATTTCGCGCATCGC	*VOA1*-F	GATAATAACCACTGCTGTGAC
BRP1-F	CGTCACCGGTCATAATTGG	*OCT1*-F	GGCGAATTTTACGTCACC
CKB1-F	GTAGTTCAAGGATTGCAAACT	*YDR114C*-F	CGTAACACAGATAGAGCAGAG
GCR2-F	CTCCTTCAAGTACCGCGAC	*YKL169C*-F	GAGTAGAACGTCAGCAGC
PRS3-F	GATTGCGAACGGTATTATTCG	*CTR1*-F	CAGATACGCAGTGTGCTC
VPS27-F	GCTATTTCTTGCGGAGCAG	*PHO85*-F	CCATAGTCCGTCCAGACAC
TCM62-F	GGCCCTTTCTTCCATTCC	*TRP2*-F	CCACAATCGATAATTAGCACTG
GSH1-F	GTTGAAGCTTGTTCTTGCCTC	*FKS1*-F	GAGGACATTAACCTCTCTTACG
POP2-F	GAATTCGGTCCTTACCCG	*SOD1*-F	GGTGTGTCGGAATTAGTAAGC
YJL175W-	GACTCACTCTTTGTGTCTCC	*YNL296W*-	CCATAGATGAACTTCCCGTATG
GSH1-F	GTTGAAGCTTGTTCTTGCC	*AFT1*-F	GAGTGATAGACGACGATTCAT
DAL81-F	GGTAGAGCAGAAGCAGTC	*OPI9*-F	CTGCAGGAACTTCAGAAGATG
LTE1-F	CATCTTTGCGATCCGATGC	*ELO3*-F	GCTCTTCACTCGCTTCAAG
TOM1-F	GAGCCGATACTAAATCACCATG	*VMA8*-F	GCATCTGTAGTACATAGGTTCC
VPS52-F	GTACACATAGGACTGCGTATC	*YEL045C*-F	CGCAACCAATACTTCTTGTTG
YAP1-F	CTTGTGCATGAACACGAG	*PEP12*-F	GACGATGAACTACTGCGC
SLT2-F	CGAACTGTGCATTCAGTCAG	*PAF1*-F	GACGACTGTAACATTGGATG
OSH3-F	CGTCCTATTTGCTGGAGAG	*LDB19*-F	GTTGAGCAACGTTATAGCTTG
ERV14-F	CCTTAATACGAAGGAGAGACC	*ANP1*-F	GCTCCATTAAACAGTTCCTTG
URE2-F	GCACTGAATTGAATCGAAGAG	*GCN5*-F	GATTGGTAAGGGAAGACCGTG
RSC1-F	CGTCAATAGTGATTGTTGTCTC	*ADA2*-F	GTACATGCCAGAGTTCGATG
CCS1-F	GCAAGGCAGAAACCCATTC		

Table S2. The protein function and subcellular localization of 64 genes sensitive to Hg^{2+}.

No.	Systemic name	Standard name	Protein function	Subcellular Localization
1	YML008C	*ERG6*	Delta(24)-sterol C-methyltransferase; converts zymosterol to fecosterol in the ergosterol biosynthetic pathway by methylating position C-24; localized to lipid particles, the plasma membrane-associated endoplasmic reticulum, and the mitochondrial outer membrane	endoplasmic reticulum (IDA)lipid droplet (IDA)
2	YPL161C	*BEM4*	Protein involved in establishment of cell polarity and bud emergence; interacts with the Rho1p small GTP-binding protein and with the Rho-type GTPase Cdc42p; involved in maintenance of proper telomere length	cytoplasm (HDA)nucleus (HDA)
3	YNL323W	*LEM3*	Membrane protein of the plasma membrane and ER; interacts specifically in vivo with the phospholipid translocase (flippase) Dnf1p; involved in translocation of phospholipids and alkylphosphocholine drugs across the plasma membrane; null mutant requires tryptophan due to mislocalization of tryptophan permease Tat2p	endoplasmic reticulum (IDA)phospholipid-translocating ATPase complex (IPI)plasma membrane (IDA)
4	YDR135C	*YCF1*	Vacuolar glutathione S-conjugate transporter; ABC-C transporter of the ATP-binding cassette family; required for vacuole fusion; forms stable complexes with vacuole fusion machinery; regulates Vam7p recruitment to vacuoles; role in detoxifying metals (Cd, Hg, As); transports GSSG that is not immediately reduced in cytosol to vacuole; transports unconjugated bilirubin, selenodigluthatione, oxidized glutathione; similar to human cystic fibrosis protein CFTR	fungal-type vacuole membrane (IDA)
5	YEL046C	*GLY1*	Threonine aldolase; catalyzes the cleavage of L-allo-threonine and L-threonine to glycine; involved in glycine biosynthesis	cytosol (IDA)nucleus (IDA)Swr1 complex (IDA)
6	YGR163W	*GTR2*	Subunit of a TORC1-stimulating GTPase and the EGO/GSE complex; subunit of Gtr1-Gtr2, a GTPase that activates TORC1 in response to amino acid stimulation; negatively regulates the GTPase cycle of Gtr1p, a Ran/TC4 homolog; subunit of EGO, a vacuolar/endosomal membrane	cytoplasm (IDA) EGO complex (IPI, IDA) fungal-type vacuole membrane (IDA) Gtr1-Gtr2 GTPase complex (IPI)

			complex that regulates exit from rapamycin-induced growth arrest and endosome to plasma membrane sorting of Gap1p; activates transcription when chromatin bound; activated by the Lst4p-Lst7p GAP complex; homolog of human RagC	late endosome membrane (IDA) nuclear chromatin (IDA) nucleus (IDA)
7	YGR214W	*RPS0A*	Ribosomal 40S subunit protein S0A; required for maturation of 18S rRNA along with Rps0Bp; deletion of either RPS0 gene reduces growth rate, deletion of both genes is lethal; homologous to human ribosomal protein SA and bacterial S2; RPS0A has a paralog, RPS0B, that arose from the whole genome duplication	cytoplasmic stress granule (IDA) cytosolic small ribosomal subunit (IDA)
8	YPL090C	*RPS6A*	Protein component of the small (40S) ribosomal subunit; homologous to mammalian ribosomal protein S6, no bacterial homolog; phosphorylated on S233 by Ypk3p in a TORC1-dependent manner, and on S232 in a TORC1/2-dependent manner by Ypk1/2/3p; RPS6A has a paralog, RPS6B, that arose from the whole genome duplication	cytosolic small ribosomal subunit (IDA) small-subunit processome (IDA)
9	YGL241W	*KAP114*	Karyopherin, responsible for nuclear import of specific proteins; cargoes include Spt15p, Sua7p, histones H2A and H2B, and Nap1p; amino terminus shows similarity to those of other importins, particularly Cse1p; localization is primarily nuclear; function is regulated by sumoylation; protein abundance increases in response to DNA replication stress	cytoplasm (IDA) nucleus (IDA)
10	YBL022C	*PIM1*	ATP-dependent Lon protease; involved in degradation of misfolded proteins in mitochondria; required for biogenesis and maintenance of mitochondria	mitochondrial matrix (IMP)
11	YGL250W	*RMR1*	Protein required for meiotic recombination and gene conversion; null mutant displays reduced PIS1 expression and growth defects on non-fermentable carbon sources and minimal media; GFP-fusion protein localizes to both cytoplasm and nucleus	cytoplasm (HDA) nucleus (HDA)
12	YBL031W	*SHE1*	Mitotic spindle protein; interacts with components of the Dam1 (DASH) complex, its effector Sli15p, and microtubule-associated protein Bim1p; also localizes to nuclear microtubules and to the bud neck in	cellular bud neck (IDA) nuclear microtubule (IDA) spindle (IDA)

			a ring-shaped structure; inhibits dynein function	
13	YGL007W	*BRP1*	Putative protein of unknown function; conserved among S. cerevisiae strains; located in the upstream region of PMA1; deletion leads to polyamine resistance due to downregulation of PMA1	cellular component unknown (ND)
14	YGL019W	*CKB1*	Beta regulatory subunit of casein kinase 2 (CK2); a Ser/Thr protein kinase with roles in cell growth and proliferation; CK2, comprised of CKA1, CKA2, CKB1 and CKB2, has many substrates including transcription factors and all RNA polymerases	protein kinase CK2 complex (IDA)<br>UTP-C complex (IDA)
15	YNL199C	*GCR2*	Transcriptional activator of genes involved in glycolysis; interacts and functions with the DNA-binding protein Gcr1p	nuclear envelope (IDA)<br>nucleus (IDA)
16	YHL011C	*PRS3*	5-phospho-ribosyl-1(alpha)-pyrophosphate synthetase; synthesizes PRPP, which is required for nucleotide, histidine, and tryptophan biosynthesis; one of five related enzymes, which are active as heteromultimeric complexes	ribose phosphate diphosphokinase complex (IDA)
17	YGR252W	*GCN5*	Catalytic subunit of ADA and SAGA histone acetyltransferase complexes; modifies N-terminal lysines on histones H2B and H3; acetylates Rsc4p, a subunit of the RSC chromatin-remodeling complex, altering replication stress tolerance; relocalizes to the cytosol in response to hypoxia; mutant displays reduced transcription elongation in the G-less-based run-on (GLRO) assay; greater involvement in repression of RNAPII-dependent transcription than in activation	Ada2/Gcn5/Ada3 transcription activator complex (IDA)<br>chromosome, centromeric region (IDA)<br>cytosol (IDA)<br>nucleus (IDA)<br>SAGA complex (IDA)<br>SLIK (SAGA-like) complex (IDA)
18	YNR006W	*VPS27*	Endosomal protein that forms a complex with Hse1p; required for recycling Golgi proteins, forming lumenal membranes and sorting ubiquitinated proteins destined for degradation; has Ubiquitin Interaction Motifs which bind ubiquitin (Ubi4p)	endosome (IDA)<br>ESCRT-0 complex (IPI)
19	YBR044C	*TCM62*	Protein involved in assembly of the succinate dehydrogenase complex; mitochondrial; putative chaperone	mitochondrial inner membrane (IDA)
20	YMR038C	*CCS1*	Copper chaperone for superoxide dismutase Sod1p; involved in oxidative stress protection; Met-X-Cys-X2-Cys motif	cytosol (IDA)<br>mitochondrial inner membrane (IDA)

			within N-terminus is involved in insertion of copper into Sod1p under conditions of copper deprivation; required for regulation of yeast copper genes in response to DNA-damaging agents; protein abundance increases in response to DNA replication stress; human homolog CCS can complement yeast ccs1 null mutant	nucleus (IDA)
21	YNR052C	*POP2*	RNase of the DEDD superfamily; subunit of the Ccr4-Not complex that mediates 3' to 5' mRNA deadenylation	CCR4-NOT core complex (IDA) cytoplasm (IDA) P-body (IDA)
22	YJL175W	*YJL175W*	Dubious open reading frame unlikely to encode a functional protein; deletion confers resistance to cisplatin, hypersensitivity to 5-fluorouracil, and growth defect at high pH with high calcium; overlaps gene for SWI3 transcription factor	
23	YJL101C	*GSH1*	Gamma glutamylcysteine synthetase; catalyzes the first step in glutathione (GSH) biosynthesis; expression induced by oxidants, cadmium, and mercury; protein abundance increases in response to DNA replication stress	intracellular (IDA)
24	YIR023W	*DAL81*	Positive regulator of genes in multiple nitrogen degradation pathways; contains DNA binding domain but does not appear to bind the dodecanucleotide sequence present in the promoter region of many genes involved in allantoin catabolism	nucleus (IC)
25	YAL024C	*LTE1*	Protein similar to GDP/GTP exchange factors; without detectable GEF activity; required for asymmetric localization of Bfa1p at daughter-directed spindle pole bodies and for mitotic exit at low temperatures	cellular bud (IDA)
26	YDR457W	*TOM1*	E3 ubiquitin ligase of the hect-domain class; has a role in mRNA export from the nucleus and may regulate transcriptional coactivators; involved in degradation of excess histones; interacts with Dia2p and is required for Dia2p degradation; required to target Cdc6p for ubiquitin-mediated destruction during G1 phase	nucleolus (HDA) nucleus (HDA)
27	YDR484W	*VPS52*	Component of the GARP (Golgi-associated retrograde protein) complex; GARP is required for the recycling of proteins from	GARP complex (IPI) Golgi apparatus (IDA)

			endosomes to the late Golgi, and for mitosis after DNA damage induced checkpoint arrest; involved in localization of actin and chitin; members of the GARP complex are Vps51p-Vps52p-Vps53p-Vps54p	
28	YML007W	*YAP1*	Basic leucine zipper (bZIP) transcription factor; required for oxidative stress tolerance; activated by H_2O_2 through the multistep formation of disulfide bonds and transit from the cytoplasm to the nucleus; Yap1p is degraded in the nucleus after the oxidative stress has passed; mediates resistance to cadmium; relative distribution to the nucleus increases upon DNA replication stress; *YAP1* has a paralog, *CAD1*, that arose from the whole genome duplication	cytoplasm (IDA)<br>nucleus (IDA)
29	YHR030C	*SLT2*	Serine/threonine MAP kinase; coordinates expression of all 19S regulatory particle assembly-chaperones (RACs) to control proteasome abundance; involved in regulating maintenance of cell wall integrity, cell cycle progression, nuclear mRNA retention in heat shock, septum assembly; required for mitophagy, pexophagy; affects recruitment of mitochondria to phagophore assembly site; plays role in adaptive response of cells to cold; regulated by the PKC1-mediated signaling pathway	cellular bud neck (IDA)<br>cellular bud tip (IDA)<br>mating projection tip (IDA)<br>mitochondrion (IDA)<br>nucleus (IDA)
30	YHR073W	*OSH3*	Member of an oxysterol-binding protein family; this family has seven members in S. cerevisiae; family members have overlapping, redundant functions in sterol metabolism and collectively perform a function essential for viability; contains FFAT motif; interacts with ER anchor Scs2p at patches at the plasma membrane; regulated by sterol binding	cortical endoplasmic reticulum (IDA)<br>cytoplasm (IDA)
31	YGL054C	*ERV14*	COPII-coated vesicle protein; involved in vesicle formation and incorporation of specific secretory cargo; required for the delivery of bud-site selection protein Axl2p and Nha1p antiporter to cell surface; related to Drosophila cornichon; ERV14 has a paralog, ERV15, that arose from the whole genome duplication	COPII-coated ER to Golgi transport vesicle (IDA)<br>endoplasmic reticulum membrane (IDA)
32	YNL229C	*URE2*	Nitrogen catabolite repression	cytoplasm (IDA)

			transcriptional regulator; inhibits GLN3 transcription in good nitrogen source; role in sequestering Gln3p and Gat1p to the cytoplasm; has glutathione peroxidase activity and can mutate to acquire GST activity; self-assembly under limited nitrogen conditions creates [URE3] prion and releases catabolite repression	
33	YGR056W	*RSC1*	Component of the RSC chromatin remodeling complex; required for expression of mid-late sporulation-specific genes; contains two essential bromodomains, a bromo-adjacent homology (BAH) domain, and an AT hook; RSC1 has a paralog, RSC2, that arose from the whole genome duplication	RSC-type complex (IDA)
34	YDR448W	*ADA2*	Transcription coactivator; component of the ADA and SAGA transcriptional adaptor/HAT (histone acetyltransferase) complexes	Ada2/Gcn5/Ada3 transcription activator complex (IDA)<br>SAGA complex (IDA)<br>SLIK (SAGA-like) complex (IDA)
35	YLR396C	*VPS33*	ATP-binding protein that is a subunit of the HOPS and CORVET complexes; essential for protein sorting, vesicle docking, and fusion at the vacuole; binds to SNARE domains	CORVET complex (IDA)<br>cytosol (IDA)<br>fungal-type vacuole (IDA)<br>fungal-type vacuole membrane (IPI, IDA)<br>HOPS complex (IPI)
36	YLR148W	*PEP3*	Component of CORVET membrane tethering complex; vacuolar peripheral membrane protein that promotes vesicular docking/fusion reactions in conjunction with SNARE proteins, required for vacuolar biogenesis	CORVET complex (IDA)<br>extrinsic component of vacuolar membrane (IDA)<br>fungal-type vacuole membrane (IDA)<br>HOPS complex (IPI)
37	YLR111W		Putative protein of unknown function; conserved across S. cerevisiae strains	cellular component unknown
38	YPL091W	*GLR1*	Cytosolic and mitochondrial glutathione oxidoreductase; converts oxidized glutathione to reduced glutathione; cytosolic Glr1p is the main determinant of the glutathione redox state of the	cytosol (IDA)<br>nucleus (IDA)

			mitochondrial intermembrane space; mitochondrial Glr1p has a role in resistance to hyperoxia; protein abundance increases in response to DNA replication stress	
39	YDL066W	*IDP1*	Mitochondrial NADP-specific isocitrate dehydrogenase; catalyzes the oxidation of isocitrate to alpha-ketoglutarate; not required for mitochondrial respiration and may function to divert alpha-ketoglutarate to biosynthetic processes	mitochondrial nucleoid (IDA)<br>mitochondrion (IDA)
40	YNL271C	*BNI1*	Formin; polarisome component; nucleates the formation of linear actin filaments, involved in cell processes such as budding and mitotic spindle orientation which require the formation of polarized actin cables; recruited to the division site in a Glc7p/Ref2p dependent manner following release of Bnr1p; functionally redundant with BNR1	actin filament (IDA)<br>cell division site (IDA)<br>cellular bud neck (IDA)<br>cellular bud tip (IDA)<br>incipient cellular bud site (IDA)<br>mating projection tip (IDA)<br>polarisome (IDA, IPI)
41	YBR126C	*TPS1*	Synthase subunit of trehalose-6-P synthase/phosphatase complex; synthesizes the storage carbohydrate trehalose, which is critically important for survival of long-term desiccation; also found in a monomeric form; expression is induced by the stress response and repressed by the Ras-cAMP pathway; protein abundance increases in response to DNA replication stress and in response to prolonged exposure to boric acid	alpha,alpha-trehalose-phosphate synthase complex (UDP-forming) (IDA, IMP, IPI)
42	YEL044W	*IES6*	Component of the INO80 chromatin remodeling complex; critical for INO80 function; involved in regulation of chromosome segregation and maintenance of normal centromeric chromatin structure; human ortholog INO80C is a member of the human INO80 complex; implicated in DNA repair based on genetic interactions with RAD52 epistasis genes	Ino80 complex (IPI)
43	YPR036W	*VMA13*	Subunit H of the V1 peripheral membrane domain of V-ATPase; part of the electrogenic proton pump found throughout the endomembrane system; serves as an activator or a structural	fungal-type vacuole membrane (IDA)<br>vacuolar proton-transporting V-type ATPase, V1 domain

			stabilizer of the V-ATPase; the V1 peripheral membrane domain of the vacuolar H+-ATPase (V-ATPase) has eight subunits	(TAS)
44	YOL076W	*MDM20*	Non-catalytic subunit of the NatB N-terminal acetyltransferase; NatB catalyzes N-acetylation of proteins with specific N-terminal sequences; involved in mitochondrial inheritance and actin assembly	
45	YDL151C	*BUD30*	Dubious open reading frame; unlikely to encode a functional protein, based on available experimental and comparative sequence data; not conserved in closely related Saccharomyces species; 96% of ORF overlaps the verified gene RPC53; diploid mutant displays a weak budding pattern phenotype in a systematic assay	cellular component unknown (ND)
46	YGR106C	*VOA1*	ER protein that functions in assembly of the V0 sector of V-ATPase; functions with other assembly factors; null mutation enhances the vacuolar ATPase (V-ATPase) deficiency of a vma21 mutant impaired in endoplasmic reticulum (ER) retrieval	endoplasmic reticulum membrane (IDA, IMP)
47	YKL134C	*OCT1*	Mitochondrial intermediate peptidase; cleaves destabilizing N-terminal residues of a subset of proteins upon import, after their cleavage by mitochondrial processing peptidase (Mas1p-Mas2p); may contribute to mitochondrial iron homeostasis	mitochondrial matrix (IDA)
48	YDR114C	*YDR114C*	Putative protein of unknown function; deletion mutant exhibits poor growth at elevated pH and calcium	cellular component unknown (ND)
49	YKL169C	*YKL169C*	Dubious open reading frame; unlikely to encode a functional protein, based on available experimental and comparative sequence data; partially overlaps the verified gene MRPL38	cellular component unknown (ND)
50	YPR124W	*CTR1*	High-affinity copper transporter of plasma membrane; mediates nearly all copper uptake under low copper conditions; transcriptionally induced at low copper levels and degraded at high copper levels; protein increases in abundance and relocalizes from nucleus to plasma membrane upon DNA replication stress; human homolog SLC31A1 can complement a yeast ctr1 ctr3 double deletion	plasma membrane (IDA)

51	YPL031C	*PHO85*	Cyclin-dependent kinase; has ten cyclin partners; involved in regulating the cellular response to nutrient levels and environmental conditions and progression through the cell cycle; human lissencephaly-associated homolog CDK5 functionally complements null mutation	cyclin-dependent protein kinase holoenzyme complex (IPI) nucleus (IDA) Pho85-Pho80 CDK-cyclin complex (IDA)
52	YER090W	*TRP2*	Anthranilate synthase; catalyzes the initial step of tryptophan biosynthesis, forms multifunctional hetero-oligomeric anthranilate synthase:indole-3-glycerol phosphate synthase enzyme complex with Trp3p	anthranilate synthase complex (IPI)
53	YLR342W	*FKS1*	Catalytic subunit of 1,3-beta-D-glucan synthase; functionally redundant with alternate catalytic subunit Gsc2p; binds to regulatory subunit Rho1p; involved in cell wall synthesis and maintenance; localizes to sites of cell wall remodeling; FKS1 has a paralog, GSC2, that arose from the whole genome duplication	1,3-beta-D-glucan synthase complex (IDA) actin cortical patch (IDA) cellular bud neck (IDA) cellular bud tip (IDA) plasma membrane (IDA)
54	YJR104C	*SOD1*	Cytosolic copper-zinc superoxide dismutase; detoxifies superoxide; stabilizes Yck1p and Yck2p kinases in glucose to repress respiration; phosphorylated by Dun1p, enters nucleus under oxidative stress to promote transcription of stress response genes; human ortholog SOD1 implicated in ALS complements a null allele; abundance increases under DNA replication stress and during exposure to boric acid; localization to mitochondrial intermembrane space is modulated by MICOS complex	cytosol (IDA) mitochondrial intermembrane space (IDA) nucleus (IDA)
55	YNL296W	*YNL296W*	Dubious open reading frame unlikely to encode a functional protein; deletion adversely affects sporulation; deletion mutant exhibits synthetic phenotype under expression of mutant huntingtin fragment, but gene does not have human ortholog	cellular component unknown (ND)
56	YGL071W	*AFT1*	Transcription factor involved in iron utilization and homeostasis; binds consensus site PyPuCACCCPu and activates transcription in response to	cytoplasm (IDA) nucleus (IDA)

			changes in iron availability; in iron-replete conditions localization is regulated by Grx3p, Grx4p, and Fra2p, and promoter binding is negatively regulated via Grx3p-Grx4p binding; AFT1 has a paralog, AFT2, that arose from the whole genome duplication; relative distribution to the nucleus increases upon DNA replication stress	
57	YLR338W	*OPI9*	Dubious open reading frame; unlikely to encode a functional protein, based on available experimental and comparative sequence data; partially overlaps the verified ORF VRP1/YLR337C	cellular component unknown (ND)
58	YLR372W	*ELO3*	Elongase; involved in fatty acid and sphingolipid biosynthesis; synthesizes very long chain 20-26-carbon fatty acids from C18-CoA primers; involved in regulation of sphingolipid biosynthesis; lethality of the elo2 elo3 double null mutation is functionally complemented by human ELOVL1 and weakly complemented by human ELOVL3 or ELOV7	endoplasmic reticulum (IDA)
59	YEL051W	*VMA8*	Subunit D of the V1 peripheral membrane domain of V-ATPase; part of the electrogenic proton pump found throughout the endomembrane system; plays a role in the coupling of proton transport and ATP hydrolysis; the V1 peripheral membrane domain of the vacuolar H+-ATPase (V-ATPase) has eight subunits	vacuolar proton-transporting V-type ATPase complex (IDA)
60	YEL045C	*YEL045C*	Dubious open reading frame; unlikely to encode a functional protein, based on available experimental and comparative sequence data; deletion gives MMS sensitivity, growth defect under alkaline conditions, less than optimal growth upon citric acid stress	
61	YOR036W	*PEP12*	Target membrane receptor (t-SNARE); for vesicular intermediates traveling between the Golgi apparatus and the vacuole; controls entry of biosynthetic, endocytic, and retrograde traffic into the prevacuolar compartment; syntaxin	endosome (IMP)<br>Golgi apparatus (IMP)
62	YBR279W	*PAF1*	Component of the Paf1p complex involved in transcription elongation; binds to and modulates the activity of RNA polymerases	Cdc73/Paf1 complex (IPI)<br>nucleus (IDA)

			I and II; required for expression of a subset of genes, including cell cycle-regulated genes; involved in SER3 repression by helping to maintain SRG1 transcription-dependent nucleosome occupancy; homolog of human PD2/hPAF1	transcriptionally active chromatin (IDA)
63	YOR322C	*LDB19*	Alpha-arrestin involved in ubiquitin-dependent endocytosis; regulates endocytosis of plasma membrane proteins by recruiting the ubiquitin ligase Rsp5p to its targets; involved in the basal internalization and turnover of alpha-factor receptor Ste2p; recruits ubiquitin ligase Rsp5p to Ste2p via its 2 PPXY motifs; inhibited by Npr1p-mediated phosphorylation, which affects translocation between the cytosol and the plasma membrane	cytosol (IDA)<br>Golgi apparatus (IDA)<br>plasma membrane (IDA)
64	YEL036C	*ANP1*	Subunit of the alpha-1,6 mannosyltransferase complex; type II membrane protein; has a role in retention of glycosyltransferases in the Golgi; involved in osmotic sensitivity and resistance to aminonitrophenyl propanediol	alpha-1,6-mannosyltransferase complex (IDA)<br>Golgi cis cisterna (IDA)

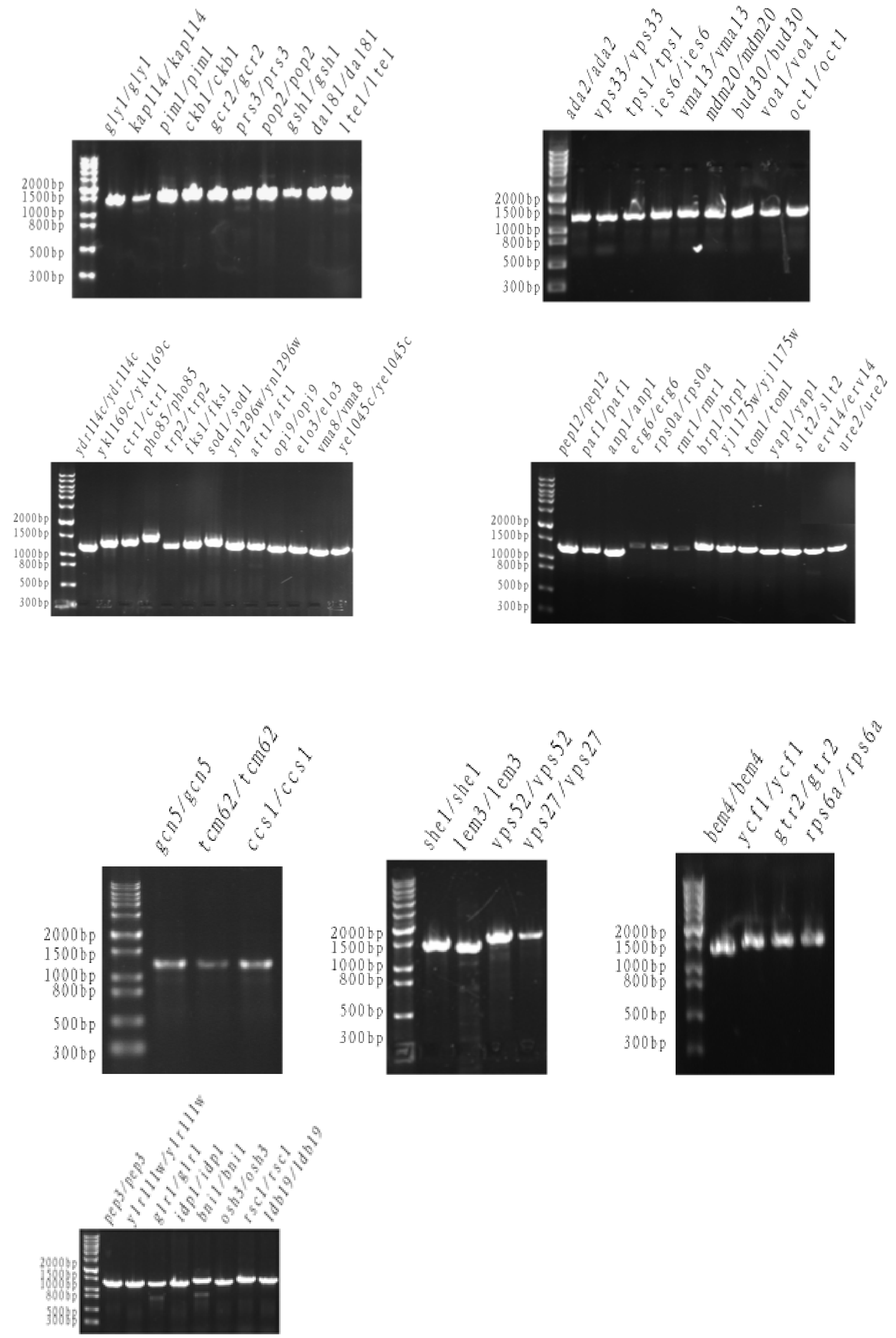

Figure S1. Genotype verification of sensitive deletion strains.
